# Can a graft be placed over a flap in complex hypospadias surgery? An experimental study in rabbits

**DOI:** 10.1590/S1677-5538.IBJU.2016.0168

**Published:** 2016

**Authors:** Ricardo Marcondes de Mattos, Sérgio R.R. de Araújo, Juliany Gomes Quitzan, Bruno Leslie, Herick Bacelar, João Luiz Gomes Parizi, Gustavo Marconi Caetano Martins, Marcela Leal da Cruz, Antonio Macedo

**Affiliations:** 1 Universidade Federal de São Paulo, SP, Brasil; 2 Universidade Estadual Paulista, Botucatu, SP, Brasil

**Keywords:** Hypospadias, Urogenital Abnormalities, Urologic Surgical Procedures

## Abstract

**Purpose:**

To develop a rabbit experimental study to test the hypothesis that surgical repair of hypospadias with severe ventral curvatures might be completed in one stage, if a graft, such as buccal mucosa, could be placed over the tunica vaginalis flap used in corporoplasty for ventral lengthening, with the addition of an onlay preputial island flap to complete the urethroplasty.

**Materials and methods:**

The experimental procedure with rabbits included a tunica vaginalis flap for reconstruction of the corpora after corporotomy, simulating a ventral lengthening operation. A buccal mucosa graft was placed directly on top of the flap, and the urethroplasty was completed with an onlay preputial island flap. Eight rabbits were divided into 4 groups, sacrificed at 2, 4, 8 and 12 weeks postoperatively, and submitted to histological evaluation.

**Results:**

We observed a large number of complications, such as fistula (75%), urinary retention (50%) and stenosis (50%). There were two deaths related to the procedure. Histological evaluation demonstrated a severe and persistent inflammatory reaction. No viable tunica vaginalis or buccal mucosa was identified.

**Conclusions:**

In this animal model, the association of a buccal mucosa graft over the tunica vaginalis flap was not successful, and resulted in complete loss of both tissues.

## INTRODUCTION

Hypospadia is one of the most common congenital deformities in humans. Its incidence varies between 1:1000 and 1:100 births (1, 2). The etiology is not fully understood, but there are theories involving testosterone deficiency, multifactorial causes, genetic predisposition, tissue remodeling and others (3). The recommended treatment is surgery at between six and twelve months of age (4).

The choice of optimal method depends on anatomical factors, one of the main challenges in surgery for severe hypospadias is the correction of severe ventral curvatures, especially in cases where only a dorsal plication is not sufficient to straighten the penis shaft, and ventral lengthening of the corpus cavernosum is necessary. A number of tissues have been used as grafts to restore corpora integrity after corporotomy; the main ones are the tunica vaginalis flap, the dermal allograft and the porcine intestinal submucosa (5).

We aimed to evaluate histologically in an animal model the hypothesis that surgical repair of hypospadias with severe ventral curvatures might be completed in one stage, if a graft, such as buccal mucosa, could be placed over the tunica vaginalis flap used in corporoplasty for ventral lengthening, with the addition of an onlay preputial island flap to complete the urethroplasty. The study focuses on assessing the dogma if grafting over a flap tissue is feasible.

## MATERIALS AND METHODS

We designed an experimental study approved by the institutional ethical review board. We treated 8 male rabbits of the species Oryctolagus cuniculus from a New Zealand strain, weighing between 2.0kg and 2.5kg. The sample was divided into 4 groups of 2 of animals, according to the date of euthanasia: sacrificed after 2, 4, 6 and 12 weeks.

### Surgical technique

We obtained a 1.0 × 0.5cm buccal mucosa graft from the donor jugal area. Using sterile techniques and a 3.5 × optical magnifying glass, the penis was freed by incising the fold between the ventral penile portion and the anus, facilitating access to the urethra. After exposing the urethra, the penile portion of it was completely separated from the corpus cavernosum, exposing the tunica albuginea. The urethra was entirely sectioned transversally, and the urethral stumps were fixed to the corpus cavernosum with 6.0 PDS suture thread, maintaining a distance of 1cm.

**Figure 1:**
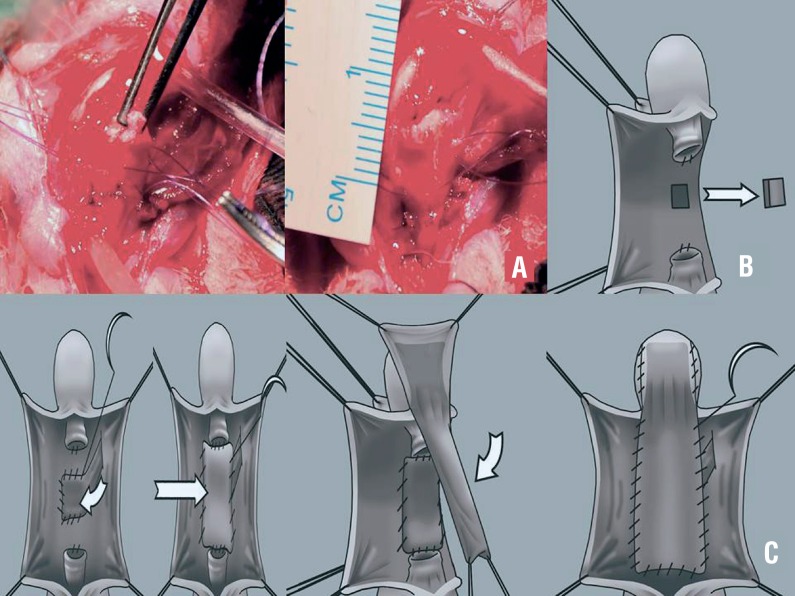
Step-by-step description of the technique consisting of a tunica vaginalis flap for reconstruction of the corpora after corporotomy. A buccal mucosa graft was placed directly on top of the flap, and the urethroplasty was completed with an onlay preputial island flap.

The left hemiscrotum (rabbits have two independent hemi scrotum) was incised and the tunica vaginalis separated from the testicle to obtain a flap that could reach the urethra. A subcutaneous tunnel was then created, so that the flap obtained could pass through the tunnel and reach the urethra. The corpus cavernosum exposed by the incision of the urethra was repaired with PDS suture 6.0, to enable repair of a defect in it, by sectioning the tunica albuginea of the corpus cavernosum over an area of 0.5 × 0.5cm, simulating a corporotomy. At this point, there was intense bleeding which was controlled by suturing the tunica vaginalis flap (visceral surface) to the edge of the defect of the corpus cavernosum with PDS 7.0, in continuous form, along three quadrants (upper, lower, and right side), keeping the pedicle of the tunica free (Figure-1). The buccal mucosa graft was then positioned over the tunica vaginalis and sutured with continuous PDS 7.0 stitches, connecting the shorter edges of the graft to the back part of the urethral stumps, thereby reconstructing the urethral plate (Figure-1). A 1.0 × 0.4cm longitudinal flap was then made out of the inner foreskin of the rabbit's penis. For this, two anchor stitches were applied to the inner foreskin, on the ventral surface, 1cm from the boundary between the skin and the mucosa. Thus, the parietal surface of the foreskin was exposed, to comprise the lumen of the future neourethra when laid over the urethral anastomosis (Figure-1). We opted to use a flap of the ventral foreskin, avoiding transposition of the flap from the dorsal to the ventral surface, simplifying and streamlining the procedure. In this way, after defining the flap, it was rotated from distal to proximal, thereby covering the urethral defect, to complete the neo urethroplasty. After rotating the flap, a continuous suture of the neourethra was made with PDS 7.0 thread. Following the urethroplasty, reconstruction of the penile skin was completed with the synthesis of subcutaneous tissue and genital skin, with separate stitches of catgut 4.0. The urethral catheter was withdrawn at the end of the procedure. Neither drains nor cystostomy were used postoperatively.

### Gross histological analysis

A macroscopic analysis of the rabbit's penis was conducted looking for scar healing, external appearance, and the presence of complications such as fistula or diverticulum. To evaluate the presence of stenosis, the urethra of each animal was catheterized with a urethral tube. The penis was sectioned at its base, preserving the corpora cavernosa and corpus spongiosum, and immediately fixed in 10% formaldehyde for a period up to 20 days.

### Microscopic analysis

**Figure 2:**
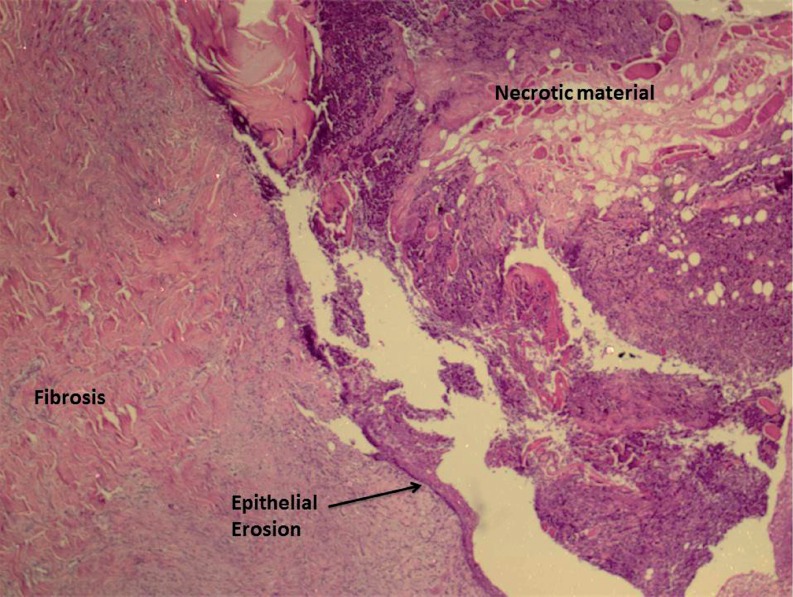
Histological aspect of the reconstructed urethra in which dorsal component consisted of a transitional urothelium and ventral component of a stratified scamous epithelium (group 8 weeks, HE × 40).

The pieces were sectioned transversally in 3mm slices, allowing the inclusion of 4 transversal segments of the same penis in each block, which were processed using the normal technique for embedding in paraffin. Slices of whole penis were included, from the glans, passing through the region that was operated on, to the base, and analyses at 40, 100, and 400 × magnification were performed with an optical microscope (Nikon Eclipse E 600). The paraffin blocks were cut to a thickness of 5 microns, with a rotary paraffin microtome, and stained with hematoxylin eosin (HE) and Masson (TM). A histological evaluation was conducted with an optical microscope by a single pathologist, specialized in the urogenital tract and experienced in the analysis of rabbit urethra (SRRA). The pathologist had no knowledge of the date of euthanasia of each case, and analyzed all the slides on the same day at the end of the study (Figure-2).

The predominant inflammatory phenomena (intensity of acute and chronic inflammation), the presence and intensity of fibrosis, the type of revetment epithelium of the neourethra, and especially, any epithelial changes that occurred postoperatively were evaluated, as well as any complications. An acute inflammatory reaction was defined as the predominant presence of polymorphonuclear leukocytes, and a chronic inflammatory reaction was defined by the predominant presence of lymphoplasmacytic infiltrate. Semiquantitative analysis of the inflammatory reaction was based on the following criteria: a score of zero for the absence of an inflammatory reaction, a score of 1 for minimal inflammation, 2 for moderate inflammation, and 3 for extensive inflammation characterized by abscesses or microabscesses for the acute type, and the presence of aggregations of lymphocytes for the chronic type. Fibrosis was characterized by the presence of fibroblasts replacing normal tissue with collagen deposits, and calculated by the distance between the urethral lumen and the beginning of the corpus cavernosum, using scores of 1 (minimal fibrosis), 2 (moderate), and 3 (maximum). The types of epithelia considered were transitional cell epithelium (urothelium), non-keratinized stratified epithelium (buccal mucosa), and keratinized stratified epithelium (lining of the inner foreskin). The complications looked for were fistulas, diverticula, stenosis, and dehiscence. The results of the histological analysis of the eight animals are shown as descriptive values and frequencies, and no statistical analysis was performed. There were no missing values to deal with.

## RESULTS

In the first postoperative week, intense hyperemia of the surgical wound and scrotal edema were observed in all eight animals. In the pair of animals sacrificed at two weeks, an increase in scrotal edema was noted, with one rabbit presenting necrosis of the scrotal skin, as well as an urethro-cutaneous fistula and inflammation of the surgical site, leading to death. The outcome of the other animal was satisfactory, and euthanasia was performed at 14 days. In the animals in Group 2 (four weeks), the presence of urethra-cutaneous fistula was noted from the second week and persisted until the time of euthanasia. However, an apparent improvement of the scrotal edema was observed.

The presence of fistula was confirmed in both rabbits in Group 3 (eight weeks). One rabbit made good initial progress, but evolved with a voluminous urethra-cutaneous fistula and died at 53 days. The animals in Group 4 (sacrificed at 12 weeks) had good postoperative evolution. One of the animals presented leakage of urine from the proximal edge of the wound during the first week, which stopped spontaneously. The other animal did not have fistula. Catheterization was not possible in four of the six animals with fistula. We consider only two of the animals, one from the 2 week group and one from the 12 week group, to have evolved without complications (Table-1). These animals were evaluated within six hours following the lethal event. Both had moisture in the genital region, indicating the presence of urethra-cutaneous fistula and difficulty inserting the tube. After removing the tube, drainage of a large amount of urine was observed, characteristic of urinary retention caused by stenosis. In the other animals, there were no difficulties with tube insertion.

**Table 1:** Description of surgical complications.

Group	Animal	Complications
**Group 1** (2 weeks)	Animal 1A	Necrosis of the scrotal skin/urethrocutaneous fistula/Death
	Animal 1B	Normal
**Group 2** (4 weeks)	Animal 2A	Urethrocutaneous fistula
	Animal 2B	Urethrocutaneous fistula
**Group 3** (8 weeks)	Animal 3A	Urethrocutaneous fistula / Death
	Animal 3B	Urethrocutaneous fistula
**Group 4** (12 weeks)	Animal 4A	Urethrocutaneous fistula (spontaneous resolution)
	Animal 4B	Normal

### Microscopic results

For a better evaluation of microscopic phenomena, the inflammatory findings, fibrosis, characteristics of the epithelium, and characteristics of the chorion are shown in Table-2.

**Table 2:** Microscopic findings of four groups in regards to inflammatory cells, fibrosis, epithelium and complications.

	2 weeks	4 weeks	8 weeks	12 weeks
Polymorphonuclear Infiltrate	2+	1 to 2+	1 to3+	1+
Fibrosis	1 to 2+	1 to 3+	2+	1+
Urethral epithelium	Stratified	Stratified	Stratified	Stratified
	Transition	Transition	Transition	Transition
	Epithelium	Epithelium	Epithelium	Epithelium
Complications	Pyocytes	Pyocytes	Fibrin crust leukocyte	Foreign body reaction
	Foreign body reaction	Granulation tissue	Ulcerations	

## DISCUSSION

Hypospadias repair is a challenging topic of urogenital reconstructive surgery, and many different techniques are currently being used (6). Around 10% of patients with proximal hypospadias have true chordees (7). In our study, we made an ambitious three concept proposal to correct complex hypospadias with: 1) dorsal buccal mucosa graft for replacement urethroplasty, ventral flap of foreskin for the ventral region of the penis and tunica vaginalis flap for corporoplasty; 2) the tunica vaginalis flap overlaying the onlay buccal mucosa graft for the urethroplasty; and 3) all this in a one-stage procedure. Two stage repair is the most commonly used technique in proximal hypospadias or in hypospadias with severe chordee, where it is necessary to section the urethral plate for correction (6, 8, 9). In fact, in cases where it is necessary to perform corporoplasty in addition to plate sectioning, there appears to be a consensus that two stage repair is preferable, with a variety of techniques used for corporoplasty (6). However, the development of a one stage procedure for complex hypospadias is still being investigated (10).

Substitution tissues possibly used in urological surgery are the arteries, veins, the ureter, the cecal appendix, the skin, bladder and buccal mucosa. Tunica vaginalis has been used extensively in surgery for the treatment of Peyronie's disease and chordee (11, 12), in the prevention of fistula and to cover suture stitches. Clinical (13) and experimental studies support the use of tunica vaginalis as a flap for ventral corporoplasty (11, 13) and urethral reconstructions, with one dorsal and one ventral “onlay” flap (14). An experimental study with rabbits comparing the tunica vaginalis as a flap with the same tissue as the graft found that the flap had good integration, but the same was not true for the graft (11). In an experimental study with rabbits in our university (15), the buccal mucosa showed good integration results. Histological results showed no fibrosis, retraction or necrosis in 12 rabbits after 6 weeks. That study inspired us to use buccal mucosa in our experiment. However, while in that study only moderate, acute inflammation was seen, we observed intense inflammation in the whole sample, with ulceration and neutrophil collections in two to four weeks. We attribute this inflammation to the surgical procedure as a whole. In 2007, Leslie et al. analyzed the use of the tunica vaginalis as a dorsal graft associated with an onlay island flap of the foreskin in urethral reconstruction surgery in rabbits, through a histopathological analysis. Histological evaluation demonstrated good incorporation of the tissues. The authors concluded that in rabbits, the histological evaluation of a tunica vaginalis graft, applied dorsally over the corpora cavernosa, combined with an onlay flap of the inner foreskin, presented total integration with the adjacent urothelium, good tolerance, and a low level of complications (fistula and diverticulum) (14).

**Figure 3:**
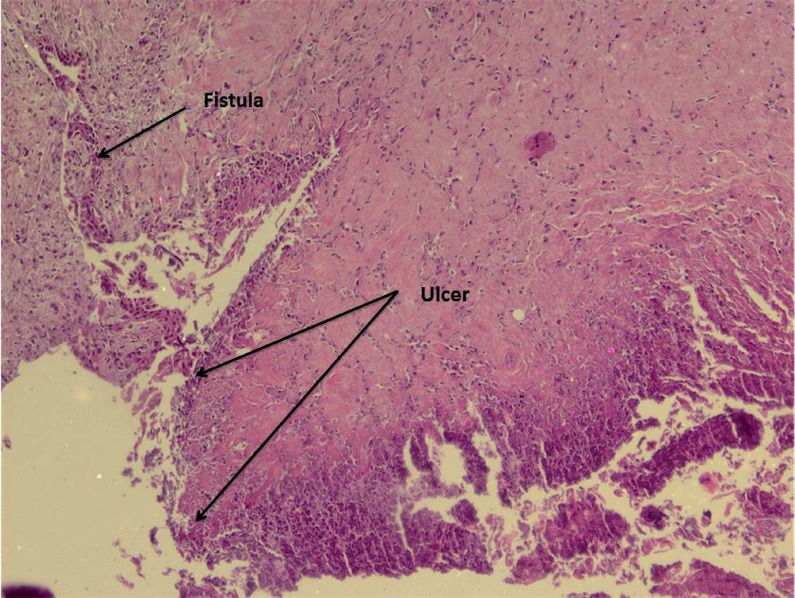
Histological representation of epithelial erosion and presence of necrotic tissue in the luminal area of the urethra (group 2 weeks, HE × 40).

The study by Leslie et al. is similar to ours in its creation of a complex surgical procedure that evaluated a dorsally applied tunica vaginalis graft, as in the analogous three-in-one technique (16). Once again it is worth noticing that even in a complex procedure, also involving a flap and a graft to perform replacement urethroplasty, we observed far fewer complications. In the animals euthanized at 12 weeks (Group 4), there was a reduction in polymorphonuclear cells and an increase in lymphoplasmacytic infiltrate. We also noted that the characteristics of the epithelium of these rabbits were entirely different from the other groups, in which acute inflammation predominated and the squamous epithelium presented ulcerations even to the point of necrosis, probably attributable to the surgical trauma as a whole (Figure-3). In 2001, Hafez et al. compared the application of the tunica vaginalis as a flap versus a graft for the correction of a defect of the tunica albuginea. In this evaluation, the flap showed evidence of an intact blood supply, viability of the cremasteric musculature without necrosis, and collagen remodeling at 12 weeks (11). In contrast, our study did not identify the tunica vaginalis in the histological analysis. We were not able to confirm the presence of the tunica vaginalis mesothelium in our slides. Thus, despite having used a technique similar to theirs, our results regarding the tunica vaginalis did not concur (11). There was no viability of the tunica vaginalis. We suggest that this must be related to the buccal mucosa graft that did not consolidate, leading to intense inflammation and loss of the flap. This study was proposed with the purpose of developing, in an animal model, a surgical technique that enabled a corporoplasty to be performed with a tunica vaginalis flap and urethroplasty with a buccal mucosa graft in a single surgery. However, we are faced with a belief in reconstructive surgery: to apply a graft directly over a flap. We did not find any studies testing this approach, and so we decided to test it.

**Figure 4:**
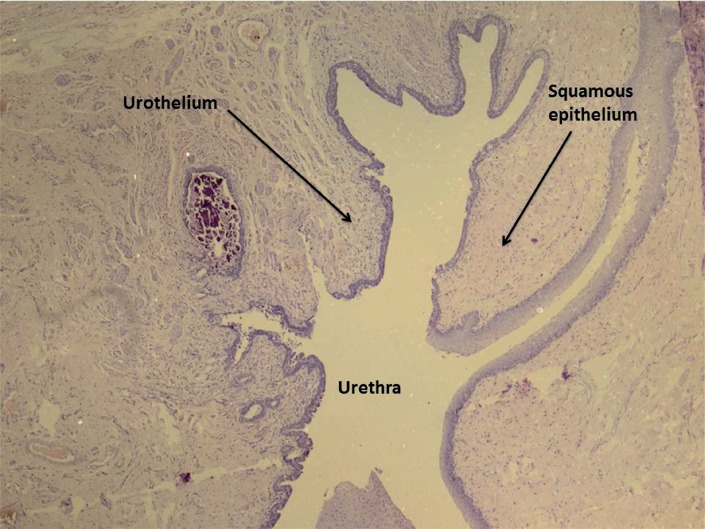
Histological aspect of urethral ulceration and fistula formation with marked inflammatory process (group 2 weeks, HE × 40).

Previous studies on hypospadias repair in our university presented very good results, with no natural deaths and two cases of fistula in the 16 rabbits in one study (14) and no complications in the other two (15, 17). In contrast, we observed two deaths (at two and eight weeks) and six animals with fistula, four of which presented urinary retention and urethra stenosis. Our complications were, therefore, more frequent and more severe. Our results were very heterogeneous among the rabbits, in terms of both clinical evolution and histological results, which we must interpret carefully. We observed unfavorable clinical outcomes overall, with all animals showing scrotal edema and intense wound hyperemia, progressing to a large number of complications. In the first week, we observed fistula in six animals (75%), three with a high volume of urine release. These data conflict with those from previous studies that involved only one tissue replacement. Histological findings confirm the unfavorable clinical findings, with a pattern of large structural changes, highlighting the absence of epithelialization and tissue regeneration, as well as intense inflammation and architectural lesions, producing fistula (Figure-4), and complicating the repair process. We also observed the formation of abscesses in four animals, with necrosis and a large amount of pyocites, mainly in the urethral lumen. The technique seemed inadequate, considering the high number of complications and unfavorable clinical outcomes. We believe that there is a multifactorial explanation for the failure of the procedure. We understand the complicating factors to be: the complexity of the surgical procedure, the extensive tissue manipulation, with prolonged surgical time, the combined use of dorsal flap and graft, the experimental defect produced in the corpus cavernosum, and the corporoplasty with urethroplasty. Which of these factors contributed to the failure of the procedure failure is something to be investigated in larger samples, with more control groups. Therefore, we recommend performing the surgery for the correction of complex hypospadias in two stages, and discourage the use of graft over flap in clinical series.

## CONCLUSIONS

The experimental model of complex hypospadias corrected with dorsal buccal mucosa graft for replacement urethroplasty, ventral flap of foreskin for the ventral region of the penis, and tunica vaginalis flap for corporoplasty; with the tunica vaginalis flap overlaying the onlay buccal mucosa graft for the urethroplasty; and one stage procedure, has resulted in a high number of complications, observed both histologically and in the clinical evolution of the experimental animals. It is not recommended that this procedure for complex hypospadias be reproduced, until the reasons for the failure have been identified.
